# A Method for Systematic Assessment of Intrinsically Disordered Protein Regions by NMR

**DOI:** 10.3390/ijms160715743

**Published:** 2015-07-10

**Authors:** Natsuko Goda, Kana Shimizu, Yohta Kuwahara, Takeshi Tenno, Tamotsu Noguchi, Takahisa Ikegami, Motonori Ota, Hidekazu Hiroaki

**Affiliations:** 1 Division of Structural Biology, Graduate School of Pharmaceutical Sciences, Nagoya University, Furo-cho, Chikusa-ku, Nagoya, Aichi 464-8601, Japan; E-Mail: tenno.natsuko@f.mbox.nagoya-u.ac.jp; 2 Computational Biology Research Center (CBRC), National Institute of Advanced Industrial Science and Technology (AIST), Tokyo Waterfront Bio-IT Research Building 2-4-7 Aomi, Koto-ku, Tokyo 135-0046, Japan; E-Mail: shimizu-kana@aist.go.jp; 3 Division of Structural Biology, Graduate School of Medicine, Kobe University, Kusunoki-cho, 7-5-1, Chuo-ku, Kobe 650-0017, Japan; E-Mail: y_kuwahara11@hotmail.com; 4 The Structural Biology Research Center and Division of Biological Science, Graduate School of Science, Nagoya University, Furo-cho, Chikusa-ku, Nagoya, Aichi 464-8601, Japan; E-Mail: tenno.takeshi@e.mbox.nagoya-u.ac.jp; 5 Pharmaceutical Education Research Center, Meiji Pharmaceutical University, 2-522-1 Noshio, Kiyose, Tokyo 204-8588, Japan; E-Mail: noguchit@my-pharm.ac.jp; 6 Institute for Protein Research, Osaka University, Yamadaoka 3-2, Suita, Osaka 565-0871, Japan; E-Mail: ikegamit@tsurumi.yokohama-cu.ac.jp; 7 Graduate School of Medical Life Science, Yokohama City University, 1-7-29 Suehiro-cho, Tsurumi-ku, Yokohama 230-0045, Japan; 8 Graduate School of Information Sciences, Nagoya University, Furo-cho, Chikusa-ku, Nagoya 464-8601, Japan; E-Mail: mota@is.nagoya-u.ac.jp

**Keywords:** solution nuclear magnetic resonance (NMR), intrinsically disordered proteins, protein flexibility, membrane protein, rotational correlation time

## Abstract

Intrinsically disordered proteins (IDPs) that lack stable conformations and are highly flexible have attracted the attention of biologists. Therefore, the development of a systematic method to identify polypeptide regions that are unstructured in solution is important. We have designed an “indirect/reflected” detection system for evaluating the physicochemical properties of IDPs using nuclear magnetic resonance (NMR). This approach employs a “chimeric membrane protein”-based method using the thermostable membrane protein PH0471. This protein contains two domains, a transmembrane helical region and a C-terminal OB (oligonucleotide/oligosaccharide binding)-fold domain (named NfeDC domain), connected by a flexible linker. NMR signals of the OB-fold domain of detergent-solubilized PH0471 are observed because of the flexibility of the linker region. In this study, the linker region was substituted with target IDPs. Fifty-three candidates were selected using the prediction tool POODLE and 35 expression vectors were constructed. Subsequently, we obtained ^15^N-labeled chimeric PH0471 proteins with 25 IDPs as linkers. The NMR spectra allowed us to classify IDPs into three categories: flexible, moderately flexible, and inflexible. The inflexible IDPs contain membrane-associating or aggregation-prone sequences. This is the first attempt to use an indirect/reflected NMR method to evaluate IDPs and can verify the predictions derived from our computational tools.

## 1. Introduction

Intrinsically disordered proteins (IDPs) are attracting the attention of biologists in terms of protein sequence–function relationships [1,2,3,4]. Structural biology methods such as X-ray crystallography and NMR spectroscopy have revealed that IDPs (including “disordered regions”) do not adopt compact-folded structures, even under physiological conditions [5,6,7,8]. The most recent biophysical view of IDPs suggests that these proteins are conformationally heterogeneous, e.g., certain secondary structures may adopt molten globules (for a review of the subject see refs. [4,9,10]). The absence of a compact fold and conformational heterogeneity are two characteristic features of IDPs, which distinguishes them from stable globular protein states. The observation that proteins function without a stable 3D structure is an emerging concept. Accordingly, many researchers believe that IDPs carry a high degree of flexibility and conformational polymorphism in their polypeptide chains.

The completion of numerous genome projects has revealed that genes encoding IDPs are present in almost all organisms, ranging from bacteria to eukaryotes [6,11]. Accordingly, IDPs have been shown to have many divergent biological functions, such as cell signaling, transcription, and translation [7,12,13,14,15]. The discovery of such proteins, which perform specific cellular roles without maintaining stable folded states, contradicts the previous model of the mechanisms underlying molecular recognition. Before IDPs gained attention in structural biology, people recognized protein–protein interactions (PPI) in the classical context of the lock-and-key model in which a rigid folded protein complementarily binds another rigid folded partner. Currently, IDPs are widely accepted because the property of IDPs can easily explain the phenomenon that conformational heterogeneity of proteins facilitates interactions with multiple targets [14]. In other words, IDPs exert specific biological roles by using their conformational flexibility and heterogeneity, which enables these proteins to modulate their binding partners according to the prevailing environmental conditions [5,16,17]. The latter issue was further studied in a PPI network associated with specific subcellular localization [18].

Prediction of IDPs from sequences is a current issue in bioinformatics, because of the rapidly increasing number of genome sequences. Based on bioinformatic analysis of genome sequences, it was concluded that the amino acid sequence of disordered regions differs from sequences of folded regions [19]. Therefore, attempts to develop prediction methods based on amino acid sequence analysis have succeeded [20,21,22,23]. Examples of the prediction tools in this category include DisEMBLE [24], GlobProt [22], PONDR, DISOPRED [20], DISpro [25], VSL2 [26], and DICHOT [27]. We have also developed a series of web applications named POODLE [28,29,30]. In designing the POODLE suite, we implemented three different programs (POODLE-S, POODLE-L, and POODLE-W) according to the length of the IDP, which is related to the function of IDPs. Among the three programs, POODLE-S was carefully designed to focus on predicting short disordered regions. This increase in the prediction accuracy was achieved by focusing on two issues: (1) amino acid propensities differ among the N-terminal, C-terminal, and internal regions [28] and (2) general physicochemical properties, rather than specific amino acids, are key factors that improve protein disorder prediction [31].

Many of the prediction tools, including our POODLE suites, were developed and fine tuned using machine learning algorithms, such as support vector machines [32]. The key step in this process is preparation of the learning datasets of amino acid sequences that are experimentally confirmed as “intrinsically disordered.” The dataset from the publicly available databases, such as DisProt [33], IDEAL [34], MobiDB [35], and pE-DB [36], containing experimentally confirmed IDPs, is particularly important for the process. The two major experimental methods to confirm the presence of disordered regions are X-ray crystallography and NMR. In case of the X-ray structures, disordered regions are identified by the lack of local electron density in the crystal. Researchers have assembled many datasets containing sequences of disordered regions from these “missing residues” in the Protein Data Bank (PDB) [33]. However, if a protein sample carries a disordered region longer than 50 residues, the protein is unlikely to crystallize. In contrast, some flexible loops within folded proteins eventually adopt a fixed conformation because of crystal contacts with neighboring molecules in the crystal, which may lead to these flexible regions being misclassified as an ordered structure. Therefore, preparing a dataset of IDPs for machine learning from crystal structures may have two opposing risks of misidentification, both under- and over-estimation. In contrast, NMR solution structures in the PDB often lack a description of “disordered” regions because of its historical definition. According to the IUPAC’s recommendation for NMR structure presentation [37], NMR structures contain multiple models of the polypeptide chain(s) in a single PDB entry without any missing residues. There are no unified criteria for extracting the IDP regions from an NMR structural ensemble. Recently, we proposed a simple assignment method to define IDP regions from the NMR-derived PDB entries [8]. Validation of NMR-refined structures, including qualification of root-mean-square deviation (r.m.s.d.) values, is another important issue. Some NMR structures tend to be “over-refined” towards well-converged structures beyond their experimental NMR constraints [38,39,40]. All of these issues may become sources of potential error during the machine-learning process of tuning IDP prediction tools. In summary, the number of examples of experimentally confirmed IDP amino acid sequences is insufficient.

**Figure 1:**
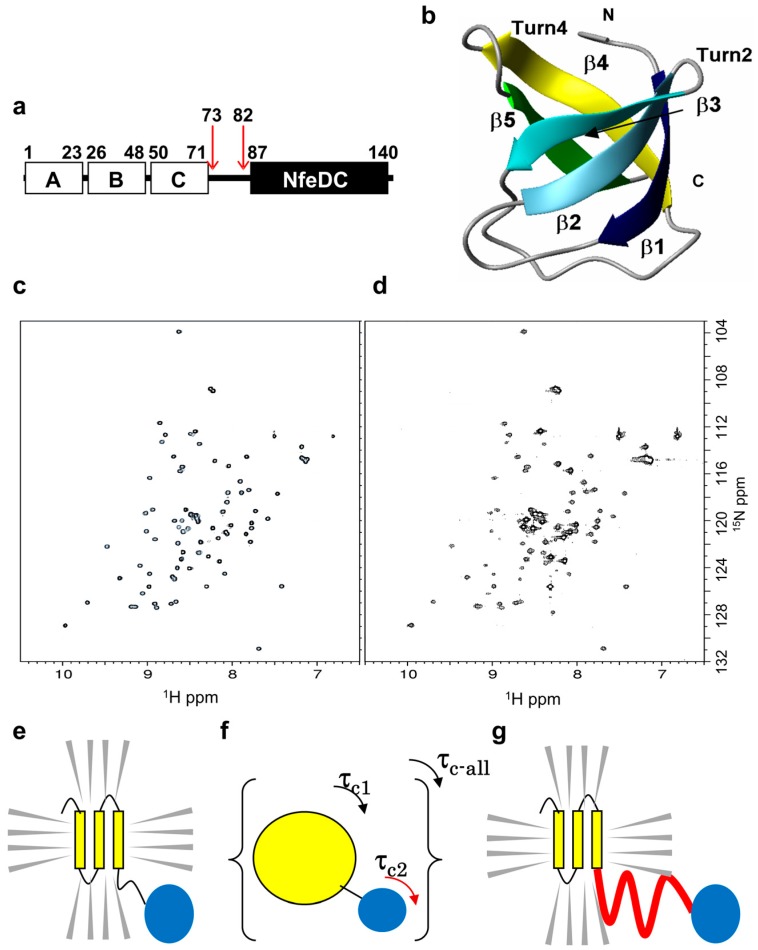
The concept and background for evaluating intrinsically disordered proteins (IDPs) using the PH0471 chimeric membrane protein-based nuclear magnetic resonance (NMR) experiment. (**a**) The domain architecture of *Pyrococcus* *horikoshii* PH0471 engineered with a C-terminal hexahistidine tag; (**b**) A ribbon representation of NfeDC^PH0471^ (PDB ID: 2exd); (**c**) The 2D ^1^H-^15^N HSQC spectrum of the isolated NfeDC^PH0471^ (NfeDC domain-only construct of PH0471); (**d**) The 2D ^1^H-^15^N HSQC spectrum of detergent solubilized full-length PH0471; (**e**,**f**) Schematics of the PH0471-based system relating to the partial (τ_c1_ and τ_c2_) and overall (τ_c-all_) molecular motion (rotational correlation time) of a two-domain system connected by a flexible linker. If the linker is rigid, the two partial τ_c_s become equal to the overall τ_c_ (τ_c1_ ≈ τ_c2_ ≈ τ_c-all_). The gray wedges and the yellow cylinders represent detergent molecules and the transmembrane helices A–C of PH0471, respectively; and (**g**) The chimeric membrane protein containing the IDP used in this study. The region indicated by a bold red line is replaced by various potential target IDPs.

There were two goals of this study, (1) to develop an NMR method suitable for the systematic assessment of the prediction of IDPs; and (2) to obtain additional physical properties of the IDPs, such as flexibility and inflexibility. For this purpose, we have designed an indirect/reflected NMR detection method of IDPs, in which we could easily assess whether the polypeptide chain was unstructured in solution in an assignment-free manner. “Assignment-free” is one of the key features of the method because making assignments is a time-consuming process. We have developed a novel “chimeric membrane protein”-based NMR method (Figure 1). In this system, we employed a “high-throughput” TA-cloning vector to express chimeric membrane proteins that carried the heterologous target IDP based on a potential restriction enzyme selectable asymmetric T (PRESAT)-vector method [41]. Finally, we evaluated 25 ^15^N-labeled chimeric membrane proteins carrying different potential IDPs that were predicted using the program POODLE. Thus, we provided a method for assessing the accuracy of the prediction tools. This assessment method is useful for verifying the prediction of IDPs and will provide a large amount of experimental data that should improve prediction methods. In addition, the method can classify IDPs into particular categories according to their flexibility. The latter feature may enable us to consider the mechanistic role of an IDP region that interconnects two globular domains, among the overall molecular function of multi-domain proteins. 

## 2. Results

### 2.1. Theoretical Background

To evaluate the physicochemical properties of IDPs by NMR, we employed a novel “chimeric membrane protein”-based method using *Pyrococcus horikoshii* PH0471. PH0471 is a thermostable, membrane-anchored, OB-fold protein, which comprises three regions: a transmembrane region (TMR) of three membrane helices (residues 1–71), a C-terminal NfeDC domain (residues 87–140), and a flexible linker that interconnects these regions (residues 73–82) (Figure 1a). In our previous study to determine the solution structure of the NfeDC domain of PH0471 (Figure 1b) [42], we observed that the NMR resonances were detected only from the NfeDC domain in a detergent-solubilized full-length PH0471 (Figure 1c,d). Full-length PH0471 in dodecyl maltoside (DDM) micelles is defined as two rigid body spheres connected by a linker (Figure 1e). In this connected-spheres system, we have defined an overall molecular rotation and a series of local molecular rotations. From NMR data, the TMR was interpreted as being incorporated into the DDM micelles and the effective molecular weight of the micelle-incorporated (protonated) TMR may exceed the detection limit of a standard two-dimensional (2D) ^1^H-^15^N heteronuclear single quantum coherence (HSQC) experiment (Figure 1d,f). In contrast, because the linker between the TMR and the NfeDC domain is sufficiently flexible, the NfeDC domain behaves independently of the whole molecular system, thereby displaying fast mobility similar to that of an isolated domain (Figure 1c,e) and NMR signals are detected for the NfeDC domain. This finding provided our initial postulate, in which researchers can indirectly evaluate the properties of IDPs using NMR signals from a domain that is connected to the potential IDP (Figure 1g). Such a reflected evaluation method is advantageous because the method does not require resonance assignments of the IDP regions that differ for each IDP sample. Sequence-specific assignment of the residues within the IDP region is not important, or to determine the 3D structure of the protein during the initial stage of a genome-wide IDP survey because IDP regions are unstructured by definition.

### 2.2. Design of the PH0471-Based PRESAT Vector

**Figure 2:**
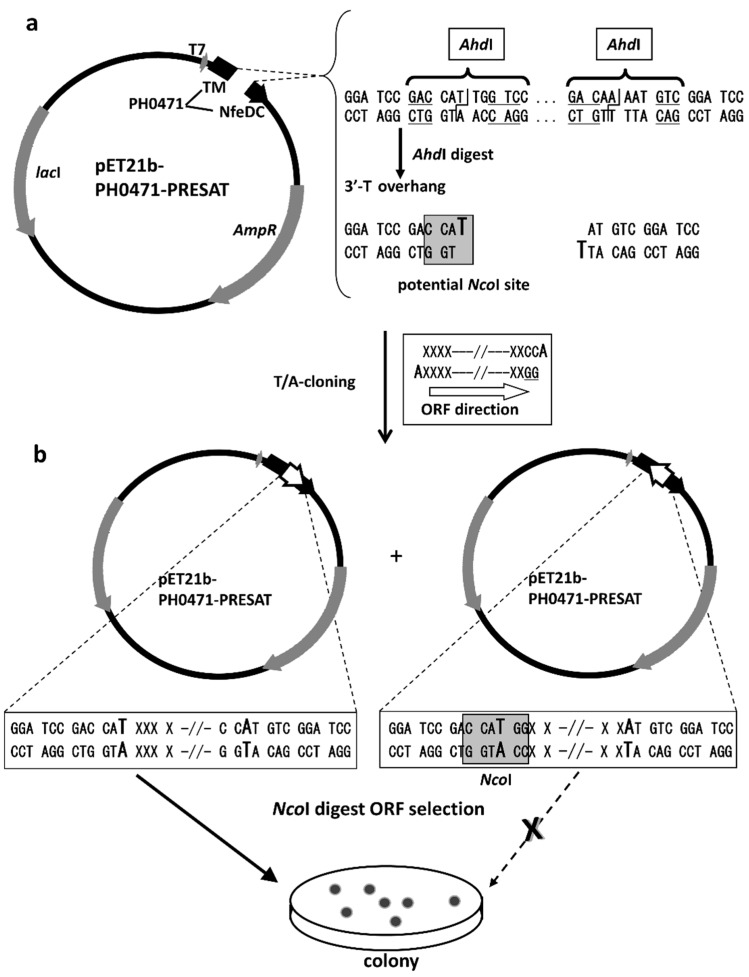
Construction of the PH0471-based PRESAT vector. (**a**) Vector map of pET21b-PH0471-PRESAT after *Ahd*I digestion; (**b**) Schematic of the unidirectional TA cloning of a PCR fragment into pET21b-PH0471-PRESAT. The gene was cloned and further subjected to ORF selection by *Nco*I digestion. The reverse PCR primer was designed to include the sequence GG at the 5′ terminus and then two 3′ A-overhangs present in the PCR products were attached by *Taq* polymerase. This ensured that only ligated plasmids with the insert in the reverse orientation have an *Nco*I site at the TA-cloning position.

Figure 2a shows the map of the PH0471-based PRESAT vector, which was designed for the experimental assessment of IDPs. The PRESAT-vector method is designed for unidirectional TA cloning that facilitates the construction of fusion protein expression vectors with limited effort and low background self-ligation. We developed several PRESAT vectors with many different linkers suited for different purposes [41,43,44]. All the vectors contained the PRESAT linker, which was designed for unidirectional TA cloning of PCR-amplified DNA fragments. The genes of interest were cloned between the two 3′ T-overhanging sites, which were produced using the restriction endonuclease *Ahd*I. These two 3′ T-overhanging sites then accept two 3′ A-overhangs in the PCR products that were attached by *Taq* polymerase. In this study, the linker was adapted to ensure that the forward (or front) and reverse (or rear) PCR cloning primers are in-frame with the TMR and NfeDC regions, respectively, without interruption by a stop codon (Figure 2b). Two additional “G” nucleotides attached to the 5′ terminal of the reverse primer enabled subsequent *Nco*I digestion when ligation occurs with the inverse (undesired) open reading frame (ORF) orientation. Thus, the site was used to select only the plasmids with the desired ORF orientation (Figure 2, bottom). When the ligation mixture was treated with *Nco*I and then transformed into *Escherichia coli*, the restriction enzyme degraded only the plasmids containing the PCR fragment in the reverse orientation, thereby eliminating unwanted transformants. All other protocols and conditions were performed according to the original PRESAT-vector method [41].

### 2.3. Proof of Method

To determine if this chimeric membrane protein-based method can evaluate IDPs, we chose a short polypeptide from a folded domain (hPCIF1 WW domain) and inserted this domain between the TMR and NfeDC domain (Supplementary Figure S1). As expected for an ordered polypeptide, the quality of the 2D ^1^H-^15^N HSQC spectrum of the NfeDC domain was poor, probably because the folded WW domain restricted the rotational diffusion of NfeDC.

### 2.4. Strategy and Workflow of the Systematic Assessment of IDPs by NMR

**Figure 3:**
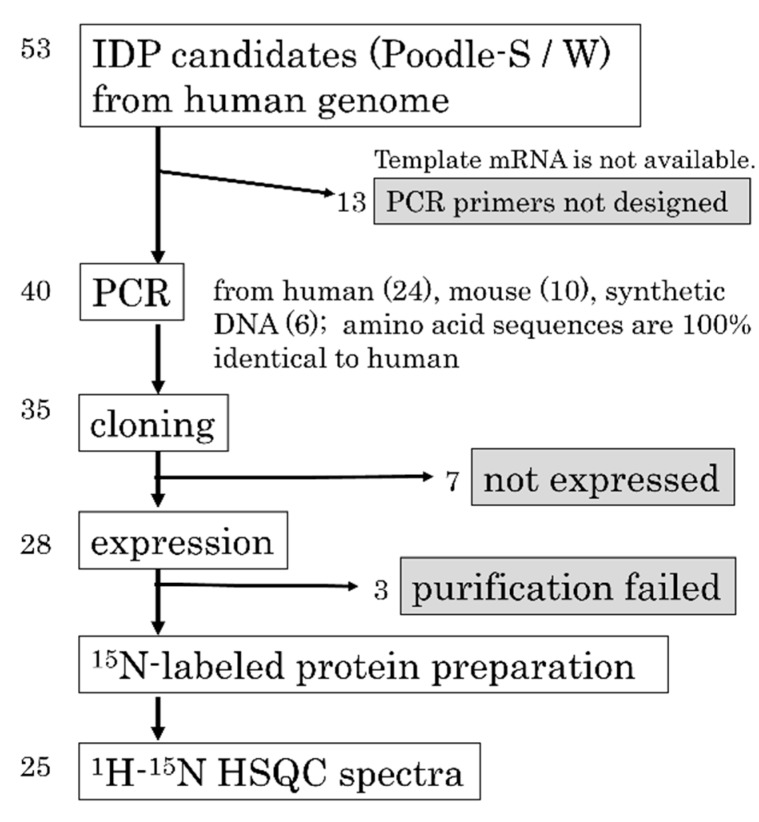
A workflow of the systematic assessment of intrinsically disordered proteins (IDPs) from the human genome.

Figure 3 illustrates the overall workflow used to assess IDPs in this study. We prepared 53 candidate sequences predicted to be IDPs using the POODLE suite. Non-redundant proteins or sections of protein that were predicted to be disordered with high probability were selected from the Human Protein Reference Database [45]. Using the annotations from the Human Protein Reference Database, we selected domains or motifs that were as many as possible and focused on the sequences from the human genome that were annotated as “structural units” rather than “unknown function” because our experimental technique was based on chimeric proteins. For experimental reasons, the length of the candidates was restricted shorter than 50 amino acids. Therefore, POODLE-S was primarily used, which is used for predicting short disordered regions. For a sequence that covers a whole protein, we also used POODLE-W to increase the reliability of the predictions. In our experiment, the candidate sequences that satisfy the following requirements were selected: (1) the candidate sequences should be a whole protein or a complete domain; (2) the length of the sequences should be shorter than 50 amino acids; (3) for a sequence that covers the whole protein, the probability score of Poodle-W should be >0.6; alternatively, Poodle-S should predict that >60% of the residues are disordered; and (4) for a sequence that covers an entire domain, Poodle-S should predict that >75% of the residues are disordered. Sequence redundancy was removed using BLASTCLUST. Because the purpose is to find novel IDPs, we removed sequences that were known to be disordered by checking the entries of DisProt [33]. Starting with 53 candidates, we succeeded in constructing 35 expression vectors. A summary of the gene accession numbers and PCR primer sequences can be found in Supplementary Table S1. We subsequently obtained ^15^N-labeled chimeric PH0471 proteins containing 25 putative IDPs as linkers. The membrane protein based-method effectively protected the samples containing IDPs from proteolytic degradation during *E. coli* expression. A summary of the UniProt entry IDs and amino acid sequences can be found in Supplementary Table S2.

### 2.5. IDPs Have Different Flexibility in Solution

**Figure 4:**
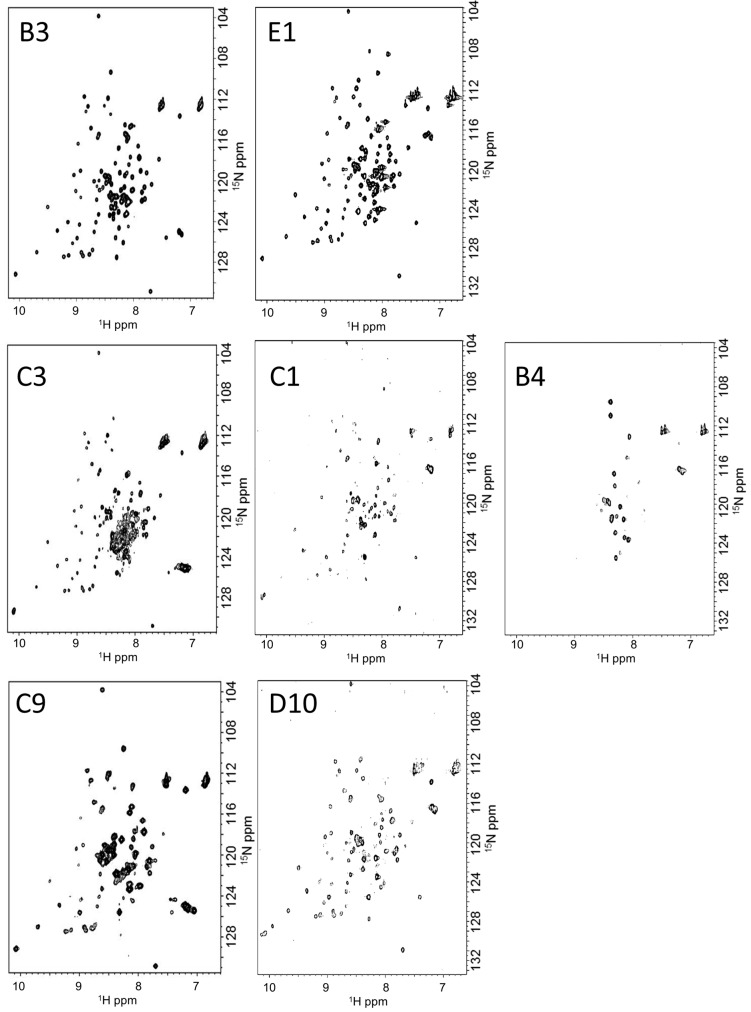
Examples of 2D ^1^H-^15^N HSQC spectra of PH0471 chimeras with intrinsically disordered proteins (IDP). (**Top panels**) Examples of IDP linkers with high flexibility; (**middle panels**) Examples of inflexible IDP linkers; (**Bottom panels**) Examples of IDP linkers with moderate flexibility.

Among the 25 putative IDPs tested, all except three gave well or moderately dispersed 2D ^1^H-^15^N HSQC resonance patterns, suggesting that these 22 predicted IDPs were disordered in solution. Of interest, we found that the NMR spectra of the chimeric PH0471 proteins with different linkers produced spectra of varying quality ranging from spectra that were missing the majority of resonances to spectra that showed excellent dispersion and a full complement of signals (Figure 4). These results clearly demonstrated that the predicted IDPs had different flexibilities in solution. For example, linkers B3 and E1 gave 2D ^1^H-^15^N HSQC spectra of excellent dispersion and equal intensities of signals, which were mostly indistinguishable from the 2D ^1^H-^15^N HSQC spectra of the soluble NfeDC^PH0471^ domain alone. However, the other 22 samples gave NMR spectra where the signals were not well dispersed, broader, and similar to the spectrum of full-length PH0471. Comparing the 2D ^1^H-^15^N HSQC spectra with two reference spectra (a negative control and a positive control, see Supplementary Figure S1), we classified the IDPs into three categories: flexible, moderately flexible, and inflexible. The inflexible IDPs C1 and B4 may represent membrane-associating or aggregation-prone sequences, which would quench flexible regions due to association. We found no example of a signal pattern that showed a fully ordered (well-folded) peptide in the linker region. Because putative IDPs were selected by the bioinformatics screening approach, the observation that only IDPs were identified by NMR verified the bioinformatics selection process. 

Our observations are the first evidence that IDPs can be classified into certain groups by their physicochemical properties, such as overall flexibility. However, this classification led us to the question “what is the biological implication of these IDP classes?” Currently, at least two distinct functions of IDPs have been proposed: “induced folding/molecular interaction coupled with folding” [14,46,47,48] and “enhanced association with target molecules by the fly-casting mechanism” [49,50,51]. The former function requires that the bound states possess a fixed conformation with specific molecular contacts after complex formation to their target proteins. The latter function may require the IDP region to have higher flexibility in its extended conformation, which interconnects two functional domains (and/or regions). Therefore, we assume that IDPs involved in “induced folding” tend to be less flexible than those involved in the “fly-casting mechanism”. We have developed a database called IDEAL (IDP with Extensive Annotations and Literature), in which experimentally verified IDPs (including regions) were collected with their involvement among the PPI network [34,52]. This database contains extensively curated IDP regions that tend to undergo induced folding upon binding to other molecules using the “coupled folding and binding” mechanism. We assumed that a certain part of these segments are less flexible than other IDP regions that do not interact with other proteins. This idea represents our next challenge to investigate using this method, e.g., investigating the potential correlation between the different physical properties of IDPs and their molecular functions.

## 3. Discussion

In this study, we developed a novel, chimeric, membrane protein-based NMR method to examine the flexibility of predicted IDPs in a high-throughput manner. This method is useful for accumulating massive datasets of experimentally proven IDP sequences, thereby contributing to the development of highly precise methods for IDP prediction. Previous methods for predicting IDPs from amino acid sequences have been developed based on experimental evidence of rigid conformations in X-ray structures deposited in the PDB. There is precedent that neither X-ray crystallography nor *in vitro* NMR methods can truly confirm whether the protein of interest is ordered or not under physiological conditions. For this purpose, in-cell NMR methods [53,54,55,56] present an ideal approach. Nevertheless, many technical difficulties exist for in-cell NMR experiments, including low sensitivity, which would hamper the accumulation of NMR data of the predicted IDPs in a high-throughput manner.

Instead, we propose that our chimeric membrane protein-based method is a powerful tool for the systematic collection of experimental data of IDPs predicted by bioinformatics tools. One of the merits of our strategy is its potency to protect the IDP samples from proteolytic degradation during bacterial expression. As we and others have experienced, bacterial expression of proteins containing unstructured regions are always challenging because of degradation by endogenous proteases [57,58,59]. The first NMR reporter parameter of structural and dynamic properties of IDPs is the amide group chemical shift. However, such an attempt is hampered by technical challenges associated with the sample preparation of IDPs. In our method, the chemical shifts from the signals of IDPs were not directly examined. Rather, we provide an assignment-free approach to assess IDPs experimentally by using an indirect/reflected NMR detection method.

The *E. coli* host cell BL21(*DE3*) used in this study, which lacks the *lon* protease, is the first choice for avoiding the problem of internal degradation; however, the use of BL21(*DE3*) cannot perfectly protect the sample. As a result, several expression systems have been developed, such as periplasmic secretion systems, thioredoxin fusion systems [43,60], and extracellular secretion systems. An alternative approach is to produce the target proteins or peptides as inclusion bodies using technologies such as N^pro^ fusion [61,62]. All these strategies choose to express the proteins in a different compartment rather than the cytosol to avoid proteolytic degradation inside *E. coli* cells. Our chimeric membrane protein-based method follows the same strategy. In addition, we designed the chimeric membrane proteins to be purified by their C-terminal hexahistidine tag to eliminate any possible digestion of the samples.

**Figure 5:**
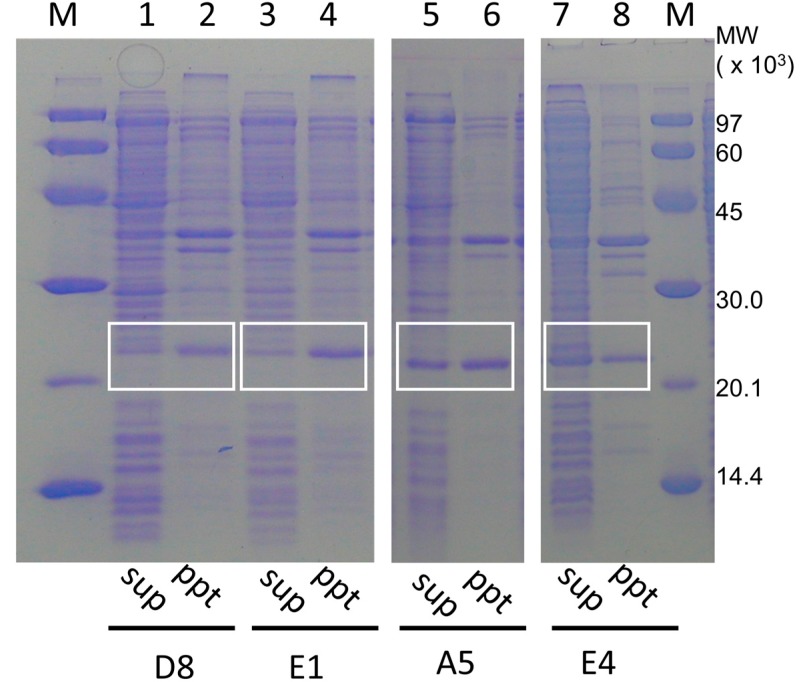
Examples of the solubility of PH0471 chimeric proteins containing various intrinsically disordered proteins (IDPs) acquired from the human genome. (Lanes **1**–**4**) Examples of the PH0471 chimera with typical solubility. (Lanes **5**–**8**) Examples of the PH0471 chimera with increased solubility. (Lanes **1**, **3**, **5**, and **7**) Proteins from the supernatant of the whole bacterial extract. (Lanes **2**, **4**, **6**, and **8**) Proteins from the pellet. (Lanes **M**, molecular weight marker). The bands containing chimeric PH0471 proteins are indicated by white boxes.

It is notable that some PH0471 fusion proteins gained solubility because of the inserted IDP sequences. Figure 5 shows the purification steps of the IDP-containing chimeric membrane proteins A5, E4, D8, and E1. The soluble and insoluble fractions of the cell lysates without detergent were analyzed by SDS-PAGE. The gain in solubility is particularly apparent for samples A5 and E4, whereas D8 and E1 were typical insoluble examples representative of all the other chimeric membrane proteins. 

Because the amino acid composition of potential IDPs are rich in charged and hydrophilic amino acids, these results are not surprising. Our result is one additional piece of experimental evidence to show that naturally occurring IDP sequences may enhance protein solubility [63], even when the IDP is incorporated into a heterogeneous protein. The flexibility of linkers A5, E4, and D8 was shown to be moderate, whereas linker E1 showed considerable flexibility, as assessed by this experimental system. Therefore, linker flexibility and enhanced protein solubility may not be directly correlated. Furthermore, our observation may partially confirm the “gate-keeper” hypothesis of Reumers *et al.* [64], in which some proteins containing amyloidogenic sequences maintain solubility by harboring a stretch of highly soluble “gate-keeper” residues adjacent to the amyloidogenic sequences.

In this study, we showed that our method facilitates classification of IDPs by their physical properties. Classification of IDPs is a state-of-the-art concept in the study of the biophysical implications of IDPs. This NMR method has clear advantages when compared with other methods, such as CD spectroscopy, which only provides information about the absence of secondary structure. We hypothesize that the physicochemical diversity among IDPs may explain their functional divergence, similar to the concept that structural diversity of folded proteins explains their functional diversity. This concept must be further strengthened by introducing more quantitative analysis of IDPs by measuring NMR parameters, such as relaxation rates and determining rotational diffusion constants. In our preliminary trial, we have introduced a protocol for semi-quantitative interpretation of the data from this method [65]. In brief, the ratio between the number of the observed HSQC resonances and the theoretical number expected in the 2D ^1^H-^15^N HSQC spectrum of the NfeDC domain-only construct was calculated. However, we should consider potential interactions between the NfeDC domain and an inserted IDP linker. The physical interaction between an IDP and its neighbor globular domain in native proteins may exhibit a critical role, such as auto-inhibition. In our method, such unexpected interactions between the target IDP and NfeDC^PH0471^, if present, can be detected as chemical shift changes to a number of the resonances in the 2D ^1^H-^15^N HSQC spectrum derived from NfeDC^PH0471^ rather than changes in signal intensities. Taken together, our method will accelerate studies of the structure–function relationships of IDPs.

## 4. Experimental Section

### 4.1. Materials

Restriction enzymes *Ahd*I, *Nco*I, *Xho*I, and *Bam*HI were obtained from New England Biolabs (Ipswich, MA, USA). T4 DNA ligase, Wizard plus SV Minipreps DNA purification kit, and a Wizard SV Gel and PCR Clean-up system (Promega, Madison, WI, USA) were used for ligation and DNA purification. Oligonucleotide primers were obtained from Hokkaido System Science Co., Ltd. (Hokkaido, Japan). The pET21b was purchased from Merck KGaA/Novagen (Darmstadt, Germany). Chelating Sepharose FF was obtained from GE Healthcare UK Ltd. (Buckinghamshire, UK). For PCR cloning, r*Taq* (Takara Bio, Inc., Shiga, Japan) was used, whereas a plasmid containing the ubiquitin gene with mutations was used as a template [43]. [^15^N]-NH_4_Cl was purchased from Cambridge Isotope Laboratories, Inc. (Andover, MA, USA). DDM was purchased from Dojindo (Kumamoto, Japan). Other biochemical reagents were purchased from Nacalai Tesque Inc. (Kyoto, Japan).

### 4.2. Vector Construction

The plasmid encoding full-length *P. horikoshii* PH0471 (143 aa) with a C-terminal hexahistidine tag under the control of the T7 promoter was used as described previously [42]. The vector contains an endogenous *Ahd*I site within the AmpR gene, which we disrupted by site-directed mutagenesis using GeneEditor (Promega), according to the manufacturer’s instructions. At the same time, we engineered two new *Bam*HI sites around amino acid residues 72 and 83 of PH0471, which are adjacent to the C-terminus of the TMR and the N-terminus of the NfeDC domain, respectively. Next, we replaced the region coding PH0471 (residues 72–83) with a linker containing the high-throughput, unidirectional TA-cloning site (PRESAT-linker) taken from pGEX-4T3-PRESAT [41], resulting in the vector pET21b-PH0471-PRESAT (Figure 2). Similar to the other PRESAT-vectors, pET21b-PH0471-PRESAT was made competent for unidirectional TA cloning after linearization using *Ahd*I, according to the procedures described by Goda *et al.* [41] In brief, 2 µg of plasmid was treated with 15 U of *Ahd*I at 37 °C for 60 min. Furthermore, using this linearized vector, we cloned more than 40 genes of intrinsically disordered and structured polypeptides, according to the protocol of Goda *et al.* [41] In brief, DNA fragments encoding putative target IDPs were prepared using either PCR or chemical synthesis. The DNA fragments encoding longer linkers were amplified by PCR using r*Taq* DNA polymerase (Takara Bio), the appropriate primers and Human *HeLa* Quick Clone-cDNA or Mouse Brain Quick Clone-cDNA (Clontech-Takara Bio) as the template (Supplementary Table S1). Synthetic DNA fragments for shorter linkers were chemically synthesized using codons optimal for *E. coli*, as shown in Supplementary Table S1. These DNA fragments were ligated and transformed into DH5α (in liquid culture). The plasmids were then recovered without colonization and subjected to digestion with the restriction enzyme *Nco*I before being transformed into BL21(*DE3*). Chimeric membrane proteins containing inserts in the desired gene direction between the TMR and NfeDC domains of PH0471 were efficiently produced using methods similar to those described previously [43], for each of the subcloning experiments (data not shown).

### 4.3. Protein Techniques

*Escherichia coli* BL21(*DE3*) cells, transformed with pET21b-PH0471-PRESAT derivatives containing potential IDPs as linkers were grown at 37 °C in modified M9 minimal medium, containing 0.5 g/L [^15^N]-NH_4_Cl as the sole nitrogen source. Expression of the recombinant proteins was induced with isopropyl β-d-1-thiogalactopyranoside (1 mM) when the cell density reached an OD_600_ of 0.4. Cells were harvested after induction for 6 h at 30 °C, and the cell extracts were analyzed by 15% SDS-PAGE. Purification of chimeric PH0471 proteins was performed using a protocol modified from the original protocol of Kuwahara *et al.* [42]. In brief, the cells were broken by sonication in lysis buffer, containing 150 mM NaCl and 50 mM Tris-HCl (pH 7.5), and centrifuged at 5000× *g* to collect the pellet fraction. The pellet was solubilized by the same buffer supplemented with 1.5% dodecyl maltoside (DDM). The proteins were purified by Ni^2+^-charged chelating-Sepharose Fast Flow. The samples were eluted using 500 mM imidazole, containing 0.1% DDM, and dialyzed with NMR buffer, containing 100 mM NaCl, 25 mM sodium phosphate buffer (pH 5.0), and 0.1% DDM, before performing NMR experiments.

### 4.4. NMR Spectroscopy

NMR experiments were performed on either a Bruker Avance DRX 500 MHz NMR spectrometer (Bruker, Karlsruhe, Germany) at Osaka University or a Bruker Avance III 600 MHz spectrometer (Bruker, Karlsruhe, Germany) at Kobe University, the latter being equipped with a cryogenic probe. ^15^N-labeled PH0471 mutants were dissolved in 0.33 mL of 90%/10% H_2_O/D_2_O containing 25 mM sodium phosphate buffer at pH 5.0, 100 mM NaCl, and 0.1% *w*/*v* DDM micelles to a final concentration of approximately 0.1 mM. 2D ^1^H-^15^N HSQC spectra [66] were acquired using gradient sensitivity enhancement [67] and the WATERGATE solvent suppression pulse scheme [68], and were acquired with 4–32 transients and 256 increments at 298 K. Datasets were zero filled during spectral processing. All two-dimensional spectra were processed with nmrPipe [69] and analyzed with the program Sparky [70].

## Supplementary Materials

Supplementary materials can be found at http://www.mdpi.com/1422-0067/16/07/15743/s1.
