# Supplementary material for: A Method for Systematic Assessment of Intrinsically Disordered Protein Regions by NMR
Source: Int J Mol Sci. 2015 Jul 10;16(7):15743–60. doi: 10.3390/ijms160715743 (PMC4519922; doi:10.3390/ijms160715743)
Supplement: Supplementary file 1 [file ijms-16-15743-s001.pdf]

Supplementary Information

Control
hPCIF1 WW domain
PDB; 2JX8

Sequence
IPEELVHAGWEKCWSRR-
ENRPYYFNRFTNQSLWE-
MPVLG

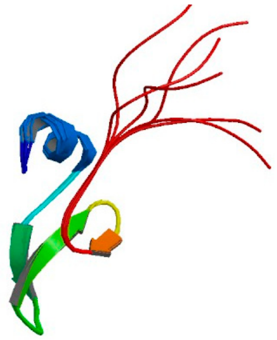

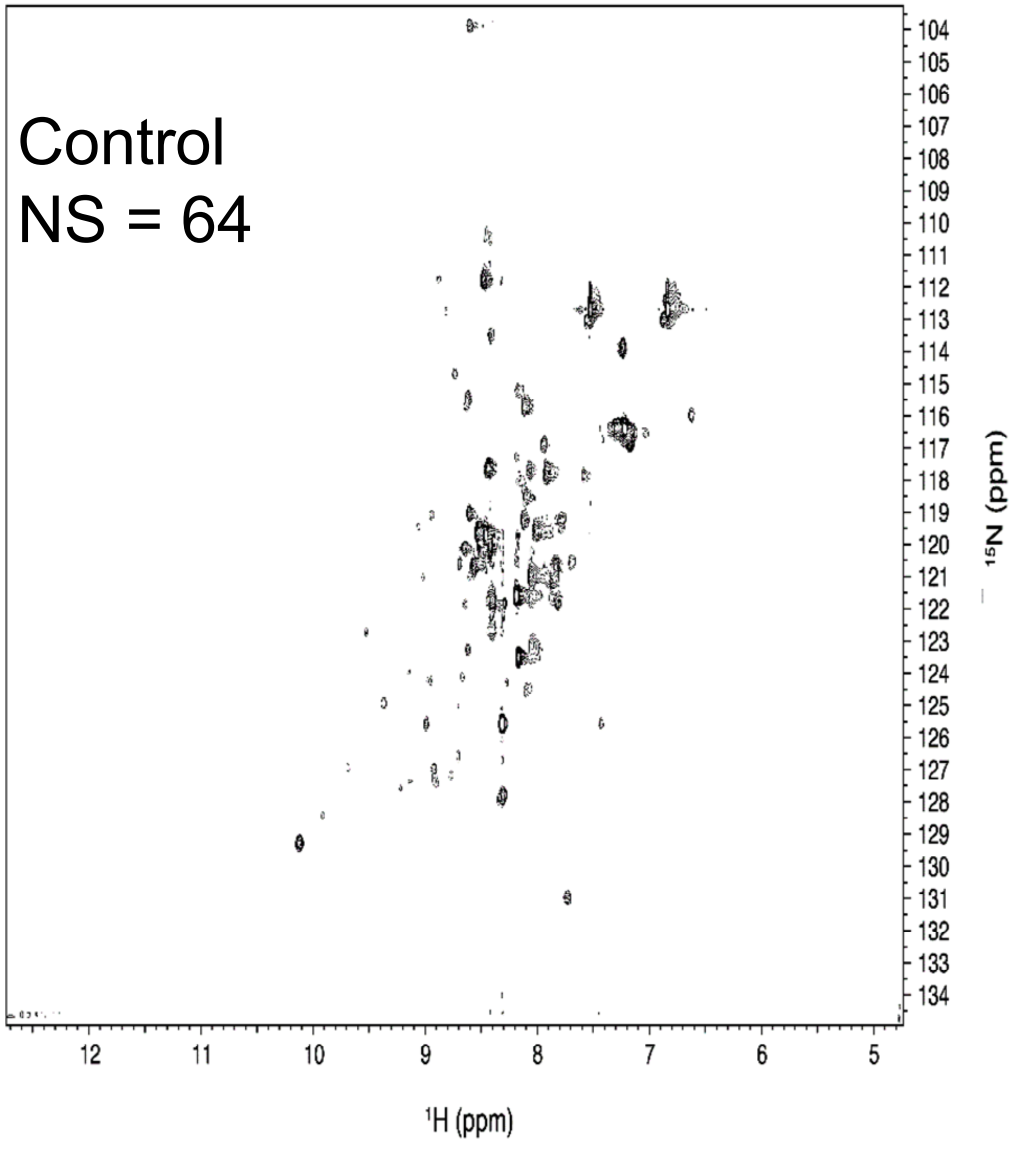

Figure S1. Examples of ^{1}H-^{15}N HSQC spectrum of PH0471 chimera with a control folded protein domain. (**Top panel**) Amino acid sequence of hPCIF1 WW domain with its ribbon diagram; (**Bottom panel**) ^{1}H-^{15}N HSQC spectrum of PH0471-hPCIF1-WW chimera (scan number = 64).

Table S1. The list of the predicted IDP genes from human genome whose genes were cloned. Gene accession numbers with front/rear PCR primer sequences are summarized.

Sample ID	Accession	Template	Front Primer	Rear Primer
A1	AAH22328.1	synthetic	aggtgttatgaatgtggagaaaagggc<br>cactatgcatatgattgtcaccgagcca	ggctcggtgacaatcatatgcatagtggcc<br>cttttctccacattcataacaccta
A2	NP_005924.2	synthetic	aggaagccacgaggacgacctcgaagcggtagtgatcgggcca	ggcccgatcactaccgcttcgaggtcgtcctcgtggcttccta
A3	NP_038286.2	synthetic	aagcgcccaccgcctccatatccaggacctggtaaggcca	ggccttaccaggtcctggatatggaggcggtgggcgctta
A4	NP_003922.1	synthetic	gcacgaagcgtattactcgaggcaatccg<br>aaagggaatccagttgcgaaaggtggcca	ggccacctttcgcaactggattccctttcg<br>gattgcctcgagtaatacgcttcgtgca
A5	NP_060834.2	synthetic	aagcacaagggacataaggagcgtcataagcaccatcatcaccgagcca	ggctcggtgatgatggtgcttatgacgctccttatgtcccttgtgctta
A6	NP_001196.1	synthetic	aaggcagagttattgcagggaggtgatttacttcgacaacgagcca	ggctcgttgtcgaagtaaatcacctccctgcaataactctgcctta
B1	AAH07821.1	HeLa	atgatcatggctcactgcag	ggcgcctcctgagtagctg
B2	NP_995312.1	placenta	atgatgaggctgccagtg	ggtgggtttgttctcagc
B3	NP_066926.1	HeLa	atggcagacaaaccagacatg	ggaaatttcactccgcttctc
B4	NP_001028678.1	HeLa	atgtcaggagacggagc	ggcaacctgtgcctctcgaatg
C1	NP_570859.1	mouse barin	atggcagctctgcgctat	ggctttggcgtagtacacccag
C2	NP_898870.1	mouse spleen	atgtctgacaaacccgatatgg	ggattcgccagcttgctt
C3	NP_066927.1	mouse brain	atgagagcgaagtggcg	ggccttggacctctgcctcat
C4	NP_002036.1	mouse brain	aaggctcataaggctgcg	ggctttgagctttttccttgttatgtgtcc
C5	NP_958782.1	mouse brain	agcctgatcttgctggaggcc	ggcgtgcagctcaggaccc
C6	NP_003160.2	mouse brain	aatgaactcataggccagactg	ggactctgtggcatcc
C7	NP_005977.2	mouse brain	agctactcgctggccggc	ggccacacccatggccacg
C8	NP_055792.1	mouse brain	agccttcaggctctgcggg	ggctagagcccctttgagccg
C9	NP_006697.2	mouse brain	agccccactgaggagatatgg	ggctccatctggcttggtccat
C10	NP_055725.2	mouse brain	gtagctctcccagagg	ggcaagggacatactgatgcctg
D1	NP_705612.1	HeLa	atgtccaggaaaccacgag	gggagagccttttgttcct
D2	AAH14919.1	HeLa	atgactcccccgcgtc	ggctctgcgctcgcgcac
D3	NP_060710.2	HeLa	gacgtgaccctgaggaa	ggctgctgtggagacgctg
D4	NP_005115.2	HeLa	gcaggcacttgggattgtgatac	ggccggtgtttcacaggctac

Table S1. *Cont.*

Sample ID	Accession	Template	Front Primer	Rear Primer
D5	NP_001012768.1	HeLa	gaaaacagtggtagcagtagt	ggccattgtgccatactgggaac
D6	NP_005091.2	HeLa	agagaaggtgtcactccctg	ggcctcatcttctttatcactttccgaag
D7	NP_998734.1	HeLa	gcatttactcccgtcgtggacg	ggcgtgagggtggaccccttata
D8	NP_006281.1	HeLa	gatgtaaaatgtgaaacgcccaactg	ggcctctgagcactcatggcataaag
D9	NP_001844.3	HeLa	gacggccaggacggt	ggccccaatggcacct
D10	NP_002537.3	HeLa	gcatttcctccaaagtaccttcattatgac	ggcgcacacggtcttccactttg
E1	NP_005222.2	HeLa	gcaggattcggcgggaagtatg	ggcttcgacaggtactgtcttctg
E2	NP_001964.2	HeLa	agccgcaatgactacatacac	ggcgttcaaagagttgagagtaaatgaag
E3	NP_004703.1	HeLa	gcacaggaggaggaggagct	ggccttctcctccgcctctg
E4	NP_002077.1	HeLa	gcatggttttttggcaaaatccccaga	ggagaagtccccaggag
E5	NP_998759.1	mouse testis	agtgacatgtacacccccgc	ggcaaagtgttgcatgtgctcatactc
E6	NP_751896.1	HeLa	gcagagacccactaccag	ggctctgttctcaatgacttcaatagatttg
E7	NP_742068.1	HeLa	gcatgtcgagatatgaagcagagag	ggcgtgtgcaaatgtacagctgg
E8	NP_055894.3	HeLa	actgtgttttataccaatgaagagtg	ggctccttcaggggtcagctt
E9	NP_006416.3	HeLa	aaagttgatttggcaaaacgaacattg	ggcctgttccactaattttcctgagg

Table S2. Summary of the putative IDPs examined in this study.

Status[1]	Sample ID	Accession	location[2]	Sequence	length
NMR	A2	NP_005924.2	173 to 185	RKPRGRPRSGSDR	13
	A3	NP_038286.2	878 to 889	KRPPPPYPGPGK	12
	A4	NP_003922.1	497 to 514	ARSVLLEAIRKGIQLRKV	18
	A5	NP_060834.2	184 to 198	KHKGHKERHKHHHHR	15
	A6	NP_001196.1	111 to 124	KAELLQGGDLLRQR	14
	B2	NP_995312.1	full	MMRLPVPSSEGPDLRGRDKRGTKEDPRADYACIAENKPT	39
	B3	NP_066926.1	full	MADKPDMGEIASFDKAKLKKTETQEKNTLPTKETIEQEKRSEIS	44
	B4	NP_001028678.1	full	MSGDGATEQAAEYVPEKVKKAEKKLEENPYDLDAWSILIREAQV	44
	C1	NP_570859.1	full	MAALRYAGLDDTDSEDELPPGWEERTTKDGWVYYAK	36
	C2	NP_898870.1	full	MSDKPDMAEIEKFDKPKLKKTETQEKNPLPSKETIEQEKQAGES	44
	C3	NP_066927.1	full	MRAKWRKKRMRRLKRKRRKMRQRSK	25
	C4	NP_002036.1	30 to 52	KAHKAATKIQASFRGHITRKKLK	23
	C6	NP_003160.2	703 to 730	NELIGQTVRISQGPYKGYIGVVKDATES	28
	C8	NP_055792.1	72 to 106	LQALRVTDLKAALEQRGLAKSGQKSALVKRLKGAL	35
	C9	NP_006697.2	132 to 164	PTEEIWVENKTPDGKVYYYNARTRESAWTKPDG	33
	D1	NP_705612.1	full	MSRKPRASSPFSNNHPSTPKRFPRQPKREKGPVKEVPGTKGSP	43
	D2	AAH14919.1	full	MTPPRPRPEEARRRPASLGHFRQGRSRPVVRERR	34
	D3	NP_060710.2	366 to 400	DVTLRKLDIKVETVNREPLTMHSDREDDTASVSTA	35
	D4	NP_005115.2	724 to 748	GTWDCDTCLVQNKPEAIKCVACETP	25
	D6	NP_005091.2	604 to 634	REGVTPWASFKKMVTPKKRVRRPSESDKEDE	31
	D7	NP_998734.1	134 to 164	FTPVVDDPVTERVRKNTQVVSDAAYKGVHPH	31
	D8	NP_006281.1	384 to 409	DVKCETPNCPFFMSVNTQPLCHECSE	26
	D10	NP_002537.3	24 to 62	FPPKYLHYDEETSHQLLCDKCPPGTYLKQHCTAKWKTVC	39
	E1	NP_005222.2	305 to 341	GFGGKYGVQKDRMDKNASTFEDVTQVSSAYQKTVPVE	37
	E4	NP_002077.1	60 to 96	WFFGKIPRAKAEEMLSKQRHDGAFLIRESESAPGDFS	37
	E5	NP_998759.1	699 to 719	SDMYTPLTTRRNSEYEHMQHF	21
exp.[3]	E7	NP_742068.1	419 to 438	CRDMKQRGGCPRGASCTFAH	20
	E9	NP_006416.3	187 to 212	KVDLAKRTLKAQKLQEELASGKLVEQ	26
not expressed	A1	AAH22328.1	full	RCYECGEKGHYAYDCHR	17
	C5	NP_958782.1	2864 to 2901	LILLEAQAASGFLLDPVRNRRLTVNEAVKEGVVGPELH	38
	D5	NP_001012768.1	250 to 287	ENSGSSSIGIPIAVPTPSPPTIGPAAPGSAPGSQYGTM	38
	D9	NP_001844.3	632 to 655	DGQDGAPGEPGPPGDPGLPGAIGA	24
	E2	NP_001964.2	137 to 156	SRNDYIHSGLYSSFTLNSLN	20
	E3	NP_004703.1	258 to 277	QEEEELQLALALSQSEAEEK	20
	E6	NP_751896.1	586 to 619	AETHYQLGLAYGYNSQYDEAVAQFSKSIEVIENR	34
only PCR	B1	AAH07821.1	full	MIMAHCSSLLGSGDPPASTSYSGG	24
	C10	NP_055725.2	565 to 587	VALPEGRQELSDGQVKTGISMSL	39
	C7	NP_005977.2	135 to 157	YSLAGGLLAAGAGGGGAAVAMGV	23
	E8	NP_055894.3	120 to 154	TVFYTNEEWELLDPTPKDLEESIVQEEKKKLTPEG	35
		NP_976056.1	full	MSRKPRASSPLSNNHPPTPKRRGSGRFPRQPGREKGPIKEVPGTKGSP	48
PCR primer not designed		AAH04343.1	full	MQQAAGFSHCLVENKPVFGASQLTSEPFTPSEGEAPKGATVDG	43
		NP_068832.1	full	MSDKPDLSEVEKFDRSKLKKTNTEEKNTLPSKETIQQEKECVQTS	45
		NP_001178.1	full	MAARAVFLALSAQLLQARLMKEESPVVSWRLEPEDGTALCFIF	43
		NP_001009956.1	full	MEEIPAQEAAGSPRVQFQSLETQSECLSPEPQFVQDTDMEQGLTGG	46
		AAM53524.1	full	MGPPGDEEPLEGPELHVLMINAPSVLAGFSNAS	33
		NP_067005.1	full	MEEQPECREEKRGSLHVWKSELVEVEDDVYLRHSSSLTYRL	41
		NP_001005414.1	full	MEAAETEAEAAALEVLAEVAGILEPVGLQEEAELPAKILVEFVVVCTR	48
		NP_851813.1	full	MDPEHAKPESSEAPSGNLKQPETAAALR	28
		NP_853651.1	full	MSFDNNYHGGQGYAKGGLGCSYGCGLSGYGYACYCPWCYERSWFSGCF	48
		NP_001008505.2	385 to 404	TNHQCLPIPSLSCWALEQGC	20
		NP_892018.1	153 to 191	RCPGNSFSCGNSQCVTKVNPECDDQEDCSDGSDEAHCEC	39
		NP_055379.1	368 to 402	GNGPSSDLSTGSSGGYPDFPASPASWLDEVDHAQF	35
		NP_443723.2	1077 to 1104	FEDIKILKEKNAELQMTLKLKEESLTKR	28

[1] Experimental status of each genes (see Figure 3); [2] For intrinsically disorder regions, there amino acid position relative to the full length proteins are indicated; [3] Expression level was too low.
